# THE IMPACT OF CLOSING MEDICARE PART D COVERAGE GAP ON MENTAL HEALTH OF OLDER ADULTS

**DOI:** 10.1093/geroni/igad104.2880

**Published:** 2023-12-21

**Authors:** Junjie Gai, Kanika Arora

**Affiliations:** University of Iowa, Iowa City, Iowa, United States; University of Iowa, Iowa City, Iowa, United States

## Abstract

The ACA mandated the gradual elimination of the Medicare prescription drug coverage gap (also called the “Doughnut Hole”) beginning in 2011. This policy change can impact mental health through mechanisms such as reduction in out-of-pocket (OOP) expenses and increased access to prescription medication. However, no previous study has examined its impact on mental health outcomes of older adults on Medicare. Employing data from the Medical Expenditure Panel Survey (from 2006 to 2016), compare the mental health of Medicare beneficiaries (66-70 years; N=7,664) with that of non-Medicare beneficiaries (60-64 years; N=8,079) before versus after policy implementation. We find that the 2011 phasing out of Medicare drug coverage gap was associated with 0.86-point (p< 0.05) improvement in the Mental Component score of the Short-Form 12 Health Survey among beneficiaries. Our analysis was robust to using alternate measures of mental health outcomes (Kessler Index and PHQ-2) as well as to an alternate construction of treatment and control groups (comparing individuals aged 60-70 years on Medicare and private insurance). We find no detectable differences in the mental health of Medicare beneficiaries who already receive subsidies for costs associated with the coverage gap through the Low Income Subsidy (LIS) program. Further, we find that elimination of the doughnut hole reduced OOP expenses by $32.65 among Medicare beneficiaries in our sample. Overall, our results for mental health and OOP expenses are mainly attributable to women. This evidence highlights indirect effects of drug coverage gap elimination on mental health outcomes among Medicare beneficiaries.

